# Epidemiological Study of Violence against Children and Its Increase during the COVID-19 Pandemic

**DOI:** 10.3390/ijerph181910061

**Published:** 2021-09-24

**Authors:** Stela Maria Tavolieri de Oliveira, Ewerton Alexandre Galdeano, Evelynne Maria Gomes Galvão da Trindade, Rafael Saad Fernandez, Rogerio Leone Buchaim, Daniela Vieira Buchaim, Marcelo Rodrigues da Cunha, Saulo Duarte Passos

**Affiliations:** 1 Pediatric Department, Jundiaí Medical School, Jundiaí 13202-550, Brazil; stelamaria@g.fmj.br (S.M.T.d.O.); ra2103025@g.fmj.br (E.A.G.); evelynnetrindade@hotmail.com (E.M.G.G.d.T.); sauloduarte@uol.com.br (S.D.P.); 2 Pediatric Emergency Service Manager, University Hospital, Jundiaí Medical School, Jundiaí 13207-450, Brazil; 3 Research Center, Sírio-Libanês Teaching and Research Institute, São Paulo 01308-060, Brazil; rafaelsaadfernandez@gmail.com; 4 Department of Biological Sciences, Bauru School of Dentistry (FOB/USP), University of São Paulo, Bauru 17012-901, Brazil; 5 Graduate Program in Anatomy of Domestic and Wild Animals, Faculty of Veterinary Medicine and Animal Science, University of São Paulo (FMVZ/USP), São Paulo 05508-270, Brazil; 6 Postgraduate Program in Structural and Functional Interactions in Rehabilitation, Postgraduate Department, University of Marilia (UNIMAR), Marília 17525-902, Brazil; danibuchaim@alumni.usp.br; 7 Department of Human Anatomy and Neuroanatomy, Medical School, University Center of Adamantina (UniFAI), Adamantina 17800-000, Brazil; 8 Morphology Department, Jundiaí Medical School, Jundiaí 13202-550, Brazil; cunhamr@hotmail.com

**Keywords:** child abuse, exposure to violence, self-destructive behavior, risk factors, emergency hospital service, COVID-19 pandemic

## Abstract

The aim of this study was to identify the epidemiological profiles of violence against children, victims, and their aggressors, and their correlations between socioeconomic and demographic factors analyzed before and during the COVID-19 pandemic. This was a cross-sectional, retrospective observational study based on a review of Individual Notification Forms from the Information System for Notifiable Diseases, including child victims of violence, under 18 years, assisted by a pediatric emergency service in Brazil, from 2016–2020. Data were stratified, then statistical analysis was performed using the two-proportion equality test and the Chi-square test, with *p* < 0.05 and a 95% confidence interval. A total of 609 notifications were analyzed and a prevalence of sexual violence (63.2%) was reported. The prevalent profile of victim was female (76.7%), aged between 2–9 years (38.1%) and 14–18 years (35.6%). The violence occurs in the victim’s home (58.9%). The prevalent profile of perpetrator was male (82.4%), young adolescent (59.2%), living as family (64%), mainly the parents (18.4%). No correlation was found between the classified socioeconomic and demographic variables and violence. There was an increase in notifications during the COVID-19 pandemic, compared to the same period in the previous year; self-harm was reported in 59.7% of physical violence in 2020. Prevalence of sexual violence was higher for females, aged between 2–9 and 14–18 years, victimized in their homes, by male offenders, living as family, mainly by their parents. No association was found between child violence and the socioeconomic and demographic.

## 1. Introduction

Abuse against children and adolescents is characterized by physical, sexual, and psychological abuse, and negligence, with consequences for their health, development, and dignity. Physical and sexual violence have high potential to cause hospitalizations, mental and functional impairment, and death [1]. Exposure to violence may result in high-risk behaviors, such as suicidal ideation, self-mutilation, chemical dependency, prostitution, anxiety, gender identity disorders, aggressiveness, compromised school and cognitive performance, and predisposition to chronic cardiac comorbidities, diabetes, cancer, and sexually transmitted diseases among children [2].

According to the World Health Organization (WHO), approximately 50% of children between ages 2 and 17 years old experience violence every year, worldwide. Moreover, 300 million children between 2 and 4 years old are victims of violent disciplinary measures, while about 120 million girls are sexually assaulted before the age of 20 [2]. Social tolerance, maintenance of family reputation, fear of social prejudice, and the inexperience of health teams result in underreporting of abuse-related cases, thus hindering the implementation of prevention measures [3,4,5] for this serious public health problem, which dates back to antiquity [6]. Countries experiencing social, economic, and health crises undergo family stress, scarcity of resources, and decreased community support, which result in higher reported rates of violence against children [7,8].

On 11 March 2020, a global crisis of pandemic proportions, caused by the SARS-CoV-2 virus, resulted in the defragmentation of social capital, family structures, and livelihoods [9,10,11]. Social distancing and quarantine measures, which were adopted to contain the spread of the pandemic, resulted in the closure of schools [12,13], home confinement, economic stress, and a change in family routine [11]. Moreover, this also led to partial temporary interruption of child abuse prevention, notification, and protection services [10]. In the same year, the WHO warned the public about greater vulnerability of children, increased incidence of abuse, interpersonal violence, and parental neglect [12]. Over 50 million cases of abuse of the vulnerable were reported by November 2020, with 1.2 million deaths, worldwide [1].

In Brazil, in 2017, there were 126,230 cases of violence against children and adolescents, with 21,559 deaths. About 25% and 10.7% of deaths occurred among children under 10 years old and those under 4 years old, respectively [10,14]. Thus, this study aimed to identify the epidemiological profile of the types of violence against children, victims, and their offenders, who were assisted in pediatric emergency services, by correlating it with socioeconomic and demographic factors and analyzing its occurrence during the COVID-19 pandemic.

## 2. Materials and Methods

### 2.1. Study Design

This cross-sectional, retrospective observational study was conducted based on the review of the Individual Notifications for Interpersonal/Self-Inflicted Violence, from the Information System for Notifiable Diseases (SINAN).

### 2.2. Study Population

The review was conducted with victims under 18 years of age, who were assisted by pediatric emergency hospital service in the state of São Paulo, Brazil, between July 2016 and December 2020. All cases of sexual, physical, and self-inflicted violence against children and adolescents, which were suspected or confirmed by clinical examination, and were family or self-reported, were included. The cases of child abuse of a psychological and negligent nature and notifications issued before the period of evaluation due to the absence of notification protocol were not considered.

The study’s emergency service belongs to the health region of Jundiaí, which is composed of 7 municipalities, with a total population of 771,000 inhabitants, 76.4% white, 18.8% brown, 3.82% black, and 0.87% Asiatic, 50.01% male and 49.99% female. With an average age of 25–39 years, the level of education of the population is divided into: no education to incomplete elementary school (41.2%), complete elementary school to incomplete high school (19.2%), complete high school up to incomplete higher education (27.5%), complete higher education (11.5%), and not informed (0.6%). As for marital status, 33.9% are married, 22.4% live in a stable relationship, 4.8% divorced, 35% single, and 3.8% widowed. Families are formed by couples without children (26.4%), couples with children (24.4%), and women without a spouse and with children (49.3%); the number of its components varies from 2 people (31.0%), 3 people (31.8%), 4 people (24.2%), 5 people (8.9%), and 6 or more people (3.9%).

### 2.3. Definitions

The present study employed the following definitions for physical and sexual abuse and self-inflicted injury:

- Physical abuse: Physical abuse refers to the intentional use of physical force with the purpose, or high probability, of causing damage to the child’s or adolescent’s health, development, survival, or dignity, possibly leading to death. These actions include beating, kicking, shaking, choking, strangling, biting, burning, and poisoning [15].
- Sexual abuse: Sexual abuse occurs when a child or teenager is subjected to sexual activity, explicit or visual, without consent, which is incompatible with their level of understanding, thus violating the laws imposed by society [8,14].
- Self-inflicted injury: Self-inflicted injury, as defined by the WHO, is a non-fatal outcome act, in which the person deliberately initiates a non-habitual behavior that, without intervention from others, will cause self-harm. This could include ingestion of a substance in excess to realize desired changes, through actual or expected physical consequences [16].

### 2.4. Ethical Procedures

This study was approved by the Research Ethics Committee of Jundiaí Medical School (protocol CAAE 23334819.8.0000.5412, opinion number 4.752.229) and used the Strengthening the Reporting of Observational Studies in Epidemiology (STROBE) development criteria.

### 2.5. Data Collection

This study identified the epidemiological profile of victims, by sex, age group according to the classification of the Notification Form (infant = ≤1 year, child = 2–9 years, pre-adolescent = 10–13 years, adolescent = 14–18 years), color, place of occurrence, and type of violence.

The offenders’ epidemiological profile, defined by Brazilian Notification Form, was also analyzed by sex, suspected drug addiction, age group (child = 0–9 years, pre-adolescent and adolescent = 10–19 years, young adult = 20–24 years, adult = 25–59 years, elderly = ≥60 years), and being a family relative or living as family with the victims.

Subsequently, socioeconomic and demographic data collected from the census tracts included the victims’ address, such as demographic area in square kilometers (km^2^) and demographic density (number of inhabitants per km^2^), number of households, economically active population (EAP), absolute population, population engaged in formal work, and average income calculated in minimum wages in real Brazilian currency.

Data from the Brazilian Institute of Geography and Statistics (IBGE) were used along with the Geofusion OnMaps^®^ software. Furthermore, they were categorized by the decile statistical criteria, obtaining five categories that are presented in Table 1.

The absolute numbers of spontaneous visits for general causes to the pediatric emergency room (ER) were collected and stratified by quarters of each of the past 5 years. The care number of violence cases were compared, quarter by quarter, with the general causes visits in the ER to characterize the historical increase in the rate of visits due to violence and its occurrence during the COVID-19 pandemic.

### 2.6. Statistical Analysis

The entire study was carried out with qualitative variables, relative frequencies and for this reason the application of the two-proportion equality test and the Chi-square test for statistical analyses. For variables with only two response levels, the *p*-value was a direct result of their comparison. As for the variables with three or more response levels, the last column in the table shows the *p*-values for the comparison of each response level, always in relation to the most prevalent that was adopted for Reference (Ref).

A significance level of *p* < 0.05 and a 95% confidence interval (CI) were adopted using R software. For the qualitative variable, age of victims, the mean age was calculated and the Student’s test (*t*-test), freedom degree, standard deviation was adopted for statistical analysis of their results. *p*-values were not adjusted for multiple testing and should be interpreted as explorative only.

## 3. Results

A total of 616 reports of physical and sexual violence were identified from July 2016–December 2020. Seven cases were excluded as they failed to meet the eligibility criteria; thus, 609 notifications were considered in this study. The mean age of the victims was 9.9 ± 5.5 years and there were two deaths caused by physical violence.

The majority of victims of violence were white (52.9%). Females comprised more than three-quarters of victims (76.7%). Of the female victims of violence, 38.1% were aged 2–9 and 35.6% were aged 14–18 years. A majority of victims of physical violence were victimized in their own homes (58.9%). Sexual abuse was found in 63.2% of the sample of victims. Approximately 85.8% of sexual abuse cases involved children (*p* < 0.01). Amongst the adolescent age group, physical violence occurred in 67.3% (*p* < 0.01) of the sample.

**Figure 1:**
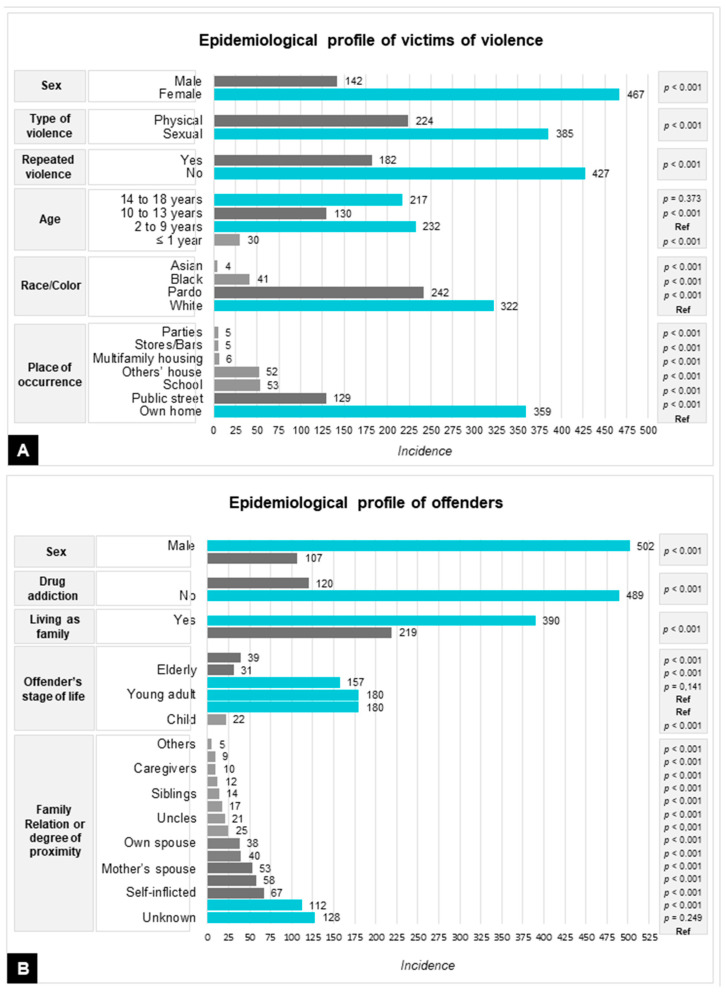
Panel of epidemiological indicators of physical and sexual violence against children and adolescents between July 2016 and December 2020. (**A**) Epidemiological profile of victims of abuse. (**B**) Epidemiological profile of offenders. (Ref) Reference category with higher incidence.

The highest prevalence among offenders was found among males (82.4%), pre-adolescents and adolescents (29.5%), and young adults (29.5%), with no suspicion of drug addiction, living as family with the victims (64%). The epidemiological profiles of victims and offenders, as well as their statistical analyses, are shown in Figure 1.

Table 2 presents the data from the complete descriptive analysis for the victim in their age groups and in the total sample (called general).

According to the data presented in Table 2, it can be noted from the general (total sample) that the average age of the victim was 9.90 ± 0.43 years, that is, a variation from 9.47 to 10.33 with 95% statistical confidence.

Table 3 presents the data comparing the victim’s gender to the average age of the victim. This analysis was performed segmented by the victim’s age group and also in the entire sample, which was called general.

There is no statistically significant difference when comparing the average age and not the age groups of male and female victims, as seen in the data presented in Table 3 (based on *p*-values). When analyzing the average age, and not the age group of the victims, there is also no statistically significant difference in relation to the type of violence suffered. In these cases, it is worth pointing out that the age range of victims is wide, ranging from a few months to 18 years.

Socioeconomic and demographic data were identified for 93.8% of the sample (Table 4).

The results of Table 4 show that there is no statistical significance between violence or the type of violence with the sociodemographic variables studied associated with the victims’ address.

Analyzing the percentage of reports of child violence showed an increase in the total number of ER visits each semester. The two semesters of 2020 show rates of violence statistically higher than the other semesters of previous years.

For physical violence, there was an increase in self-injury in the year of the COVID-19 pandemic (*p* < 0.01), corresponding to 59.7% of the 67 cases identified throughout the analyzed period. The rate of self-inflicted violence from the second semester of 2016 (0.003%) to the second semester of 2019 (0.025%) is statistically equal throughout the period. However, in the first half of 2020 the index rose to 0.117% and in the second half of 2020 it reached 0.156% (Supplementary file—Figure S1). According to the *p*-values in Table 4, it is observed that the two semiannual indices for 2020 are statistically different from all other indices for the other periods; with 11.7 and 15.6 self-inflicted injuries for every 10,000 cases, in the two semesters of 2020, respectively.

**Figure 2:**
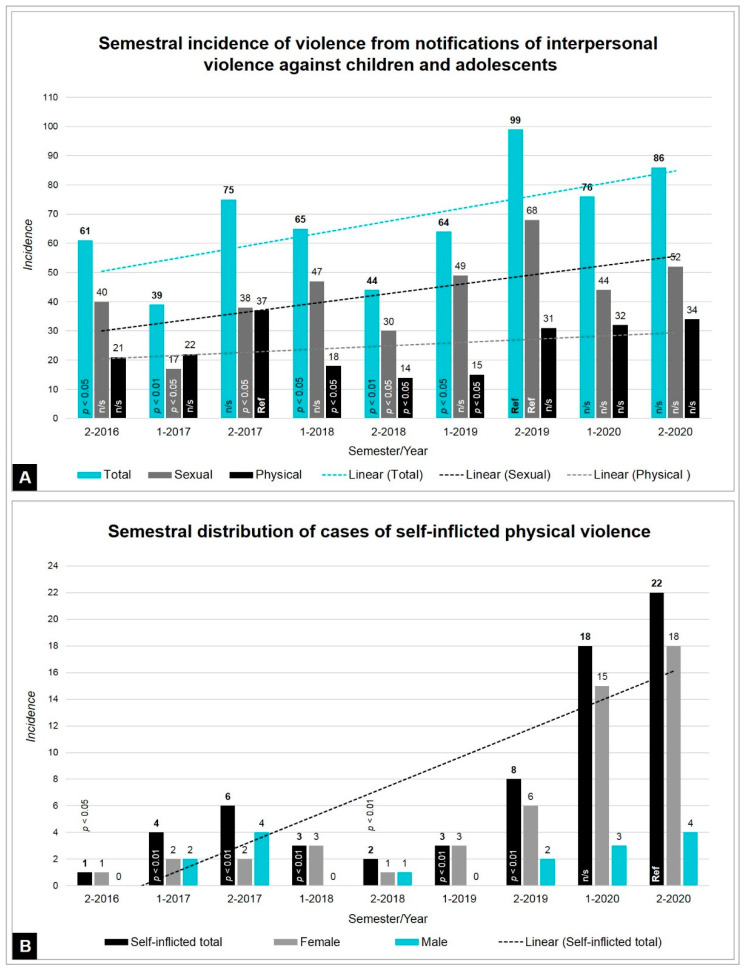
Indicators of the semestral epidemiological increase of violence. (**A**) Semester absolute totals and stratification by sexual and physical types. (**B**) Semestral incidence of self-inflicted violence stratified by sex of victims.

Accounting for 29.9% of the physical violence consultations, self-inflicted violence was prevalent in female adolescents, corresponding to 76.1% of self-inflicted notifications (*p* < 0.01), whose mean age was 14.8 ± 2.7 years, as shown in Figure 2.

The self-injury methods were drug intoxication (61.2%), self-mutilation (17.9%), poisoning (11.9%), illicit drug overdose (3%), hanging (3%), and falling (3%). Self-inflicted violence was most prevalent in female adolescents (76.1%; *p* < 0.01).

**Figure 3:**
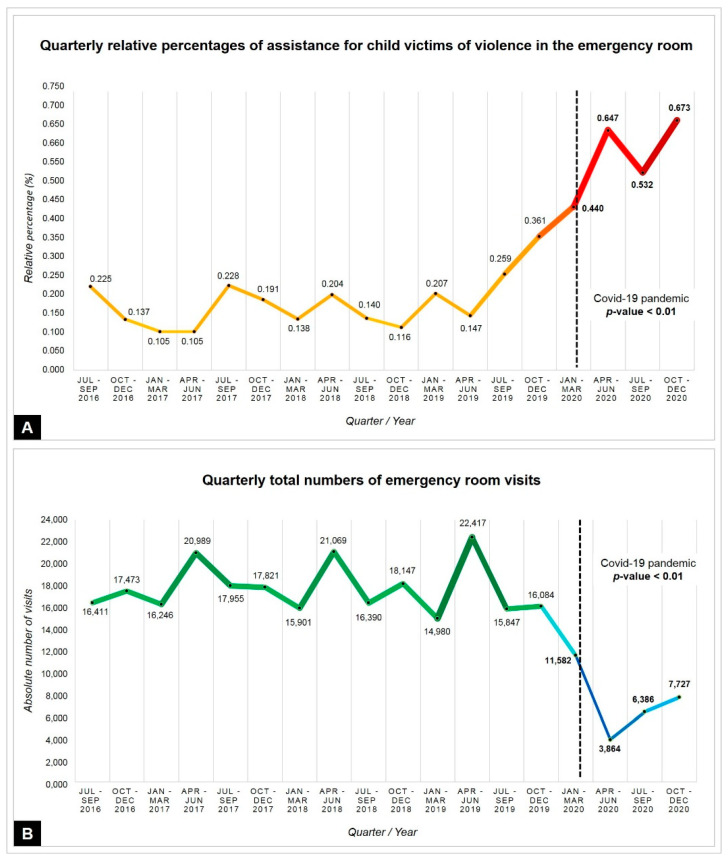
Indicators of emergency room visits. (**A**) Quarterly relative percentage of the number of visits due to violence; in red color and thick line, representation after the start of the pandemic by COVID-19. (**B**) Absolute numbers of emergency room visits; in blue color and thin line, representation after the start of the pandemic by COVID-19.

The quarterly stratification of total ER visits and reports of child violence showed inversely proportional incidences during the pandemic. During quarantine, there was less demand for medical assistance for general causes when compared with previous years; however, there was an increase in the relative percentage of assistance due to violence compared with previous quarters (*p* < 0.01), as shown in Figure 3.

## 4. Discussion

Physical and sexual abuse is a serious public health problem, with long-term repercussions for society and the economy, since it results in emotional consequences, mental illness, cognitive deficits, and suicidal ideation. Moreover, measures such as social isolation, confinement, and closure of schools that have been implemented to contain the spread of the pandemic may have led to an increase in violence against children [2]. The present study reported an increasing incidence of cases of sexual, physical, and self-inflicted violence in recent years. The results corroborate the epidemiological report of the Ministry of Health (MH) of Brazil, revealing an 83% increase in violence between 2011 and 2017. Furthermore, the highest prevalence of sexual violence occurred with female victims (74.2–92.4%), in the domestic environment (69.2%) by male offenders (83.7%). However, in contrast, a predominance of black victims was observed in 45.5–55.5% of notifications registered in Brazil [17].

This study identified a predominance of white victims, which can be explained by the regional history of colonization by European immigrants. This study reported 385 cases of sexual violence in less than five years, corresponding to 0.21% of the total number of notifications registered in Brazil in a seven-year period, prior to the pandemic [17]. The fact that children and adolescents face a greater risk of physical, sexual, and emotional abuse within their homes and by their parents [2,10,18] may have its roots in the historical patriarchal culture, with an adherence to the Code of Hammurabi, as well as the disposal of disabled children, supremacy by force, and exploitation of women [6,13]. Moreover, adults who experienced violence in their childhood have a 70% chance of repeating this behavior with their children and partners [19].

Mapping domestic violence is challenging because it takes place in a restricted, family environment, where economic dependence, physical weakness, lack of awareness, and limited capacity for manifestation strengthen the law of silence, thus benefiting the offender [3,7,20]. During the pandemic, this violence was reinforced by social isolation, restriction of movement, working from home, remote classes, closure of public spaces, schools, churches, and parks, and the online operation of Guardianship Councils [5,18]. Older children and those with more resources are more likely to seek help by contacting the Human Rights Hotline (Disque 100), 190 (Military Police), 192-SAMU (Mobile Emergency Service), or through social media [13]. Many countries do not have indicators and support hotlines, which are fundamental components of child protection systems that help to understand the country-wise epidemiology of violence [2,21].

A study from Child Helpline International, whose databases account for 41% of the world population data of children and adolescents, registered an increase from 7% to 16% of calls reporting violence through the COVID-19 helplines during the first half of 2020. In France, there was a 34% increase in reports of children exposed to dangerous situations, while in Argentina, in April 2020, there was a 67% increase in requests for help from victims of abuse [22]. In Brazil, in July 2020, Law No. 14,022 was published to ensure the maintenance and full functioning of all systems that dealt with domestic violence against women, children, and adolescents, during the pandemic. During this period, reports on violence against children increased 7.4% to 73% in different districts from north to south. Only in the State of Santa Catarina, in southern Brazil, was there an unexpected reduction of 55.3% of notifications during the isolation period, due to the difficulties encountered in seeking institutions for protection and assistance. Therefore, the complex Brazilian epidemiological profile could result in underreporting of cases [10].

Cases of violence can be underreported mainly due to absence from school activities during quarantine [2,3,5,10]. Teachers are fundamental to the surveillance of child abuse [20]. Moreover, those in already vulnerable situations are facing higher risk due to the pandemic [10]. According to the United Nations Educational, Scientific and Cultural Organization (UNESCO), approximately 1.5 billion children and adolescents were affected due to the closure of schools [2,5]; moreover, according to the United Nations Children’s Fund (UNICEF), 1.8 billion children live in countries where child abuse prevention, reporting, and protection services have been temporarily interrupted [1].

This study also analyzed the possible associations between the incidence of violence against children and socioeconomic and demographic aspects obtained from the victims’ addresses. However, we did not identify any significant association or evidence of the possible risks of child victimization due to environmental conditions [21]. This result contrasts with a study conducted in Spain, which evaluated the rates of child abuse in 552 census sectors and reported a significant correlation with low economic and educational levels [20]. A recent meta-analysis indicated a possible influence of intergenerational, cultural, and psychological backgrounds in the perpetration of child abuse [23]. The greater vulnerability of the female child population to sexual violence found in this study is corroborated by epidemiological profiles described in existing literature [3,14,23]. Previous studies also confirm this finding, reporting 60% [24] and 84% [2] of cases of violence registered among girls in Uruguay and Nigeria, respectively.

The experience of abuse in childhood, especially physical and sexual, can have adverse psychological and intellectual effects on victims, which can be manifested through anxiety disorders, depression, self-destructive behavior, and suicidal ideation later in life [3,9]. The negative effects on mental health can further deteriorate during social confinement, with the exacerbation of psychological disorders [2,11]. Experiences with other forms of violence can result in self-destructive behavior; thus, prevention, support, and protection measures are needed to avoid victimization, especially in situations of social confinement, such as the COVID-19 pandemic [13,25].

The analysis of data prior to the pandemic found a slight increase in the incidence of sexual and physical violence in children and adolescents, who were treated in emergency rooms. As of March 2020, the total number of cases by spontaneous demand decreased 42.6%, possibly due to fear of COVID-19 exposure in the hospital environment. However, the rate of assistance due to child violence, which ranged from 0.105–0.361% from 2016–2019, reached a rate of 0.673% in 2020. The two reported deaths were caused by physical violence before the pandemic period. The deleterious effects of social isolation on mental health and family well-being are known. For instance, after the Loma Prieta earthquake (1989), the Hugo (1989) and Floyd (1999) hurricanes in Central and North America, and during the Ebola virus epidemic (from 2014–2016) in West Africa, considerable increases in rates of intra-family violence were identified [5].

In terms of mental health, this study found a significant increase in cases of self-harm, which accounted for 60.6% of physical aggression in 2020. Similar data were identified in England, suggesting a negative effect of confinement and psychological impairment. In both studies, the predominant form of self-harm was drug intoxication. Self-inflicted injuries represented 29.9% of the total number of visits due to physical violence during the study period. Moreover, self-harm was most prevalent in females, with a mean age of 14.8 years; adolescents showed a propensity for emotional and mental disorders, expressing help-seeking behavior to relieve other forms of pain, which may indicate recurrent suffering [16]. These results are corroborated by the latest report of Violence and Accident Surveillance (Viva) of the Ministry of Health of Brazil (2019), which showed a 165% increase in interpersonal/self-inflicted violence between 2011 and 2017. People under the age of 19 years represented 24.4% of the total number of cases, while self-harm inflicted by this age group accounted for 30.6% of the total age group [26].

The present epidemiological analysis showed that sexual violence was predominantly targeted at females, children aged 2–9 years, and adolescents aged 14–18 years, who were assaulted in their homes, mainly by their parents. However, the incidence of sexual abuse decreased from 71.8% in 2019 to 59.3% in 2020. Based on data from 30 countries, the UNICEF declared that only 1% of adolescent victims of sexual violence in childhood sought assistance through health services. The Violence Against Children Survey from six countries found that the prevalence of formal help-seeking behavior did not exceed 28% of the victims. Self-blame, apathy, lack of need, and willingness to seek assistance services are some of the reasons for this low number [22].

The age groups of victims and offenders were distributed based on data obtained from the Notification Forms. For victims, the age is exact, as they are being attended to at the Emergency Room, at the same time the Notification is filled out. Thus, it becomes possible to classify them according to the most specific age group in infant (≤1 year), children (2–9 years), pre-adolescents (10–13 years), and adolescents (14–18 years). Regarding the age of the offenders, it is informed from the point of view of the victim or his/her guardian, under an important emotional effect and during medical care. The victim informs the age that he believes to be the closest to the real one, as the offender almost never accompanies the victim to the Emergency Room. Many families do not know the correct age of their relatives and acquaintances. In the case of a suspected age of the offender, a broader and more approximate age range was used: child (0–9 years), adolescent (10–19 years), young (20–24 years), adults (25–59 years), elderly (≥60 years), and not informed.

A recent publication cautioned that children and adolescents were more vulnerable to sexual violence during the pandemic. Moreover, sexual abuse most negatively and devastating effects on the victims’ mental, psychological, and intellectual health, and may be manifested in future stages of life, such as anxiety disorders, depression, self-destructive behavior, and suicidal ideation [3,9]. Economic impact, social isolation, and fear of exposure to COVID-19 [16] may have influenced a decreased number of reports on currents sexual violence [27].

The present study found repeated violence in 30% of cases. Adults who suffered violence in their childhood have a 70% chance of repeating this behavior with their children and partners [19]. Physical abuse in childhood has a cumulative effect according to intensity and duration of exposure, thus increasing the risk of psychosocial retardation in adulthood [23]. Approximately 10% of child hospitalizations due to burns [28] are commonly associated with ill-treatment, presence of violent parents, or households with people that do not belong to the family [15].

Children who witness or live with mothers who suffer domestic violence are at high risk of suffering aggression [22], especially traumatic brain injury (TBI), which is the most common cause of hospitalizations and deaths [1]. The United Kingdom found an alarming increase in neurological damage, associated with pandemic-related restrictions. In London, from March–April 2020, the number of hospital admissions for TBI and central nervous system bleeding increased from 0.67 to 10 cases per month [29].

Prior literature considers drug addiction as a risk factor for violence against children [2]. The present study was not able to identify this association in the analyzed sample, as it included subjective data of victims at the time of notification. Other limitations of this study were the failure to establish correlations between self-harm and reports of other episodes of abuse. Moreover, educational level, pregnancy, physical and mental disabilities, intergenerational history of violence, family stress, cultural plurality, and criminality, which may influence epidemiological rates, were not evaluated.

The scarcity of records on child violence makes it difficult to understand the epidemiological evolution of this public health problem and its correlation with COVID-19-related restrictions. Therefore, it was impossible to determine whether the pandemic has increased the frequency and severity of the usual instances of violence, or whether it has caused an increase in new cases [30]. For 30 years, the Brazilian Child and Adolescent Statute (ECA) has been providing legal support for the rights to comprehensive health, physical and emotional development, education, dignity, and a life with no violence, oppression, or discrimination. It confers on society the duty to enforce these rights and impose penalties on those who transgress them [4,7,10]. However, societies carry infanticide in their biographies, as well as a contempt for childhood, imposing supremacy, and force [6].

## 5. Conclusions

This study concluded that parents and family members were responsible for most cases of violence against children and adolescents. The incidence of violence against children has witnessed a growing trend in recent years, especially sexual abuse. The prevalent profile of the victim was female, white, aged 2–9 years or 14–18 years, and sexually abused at home. The offender’s profile was male, pre-adolescent, adolescent or young adult, with no suspicion of drug addiction, living with the victim; the main kinship was predominantly the father.

No associations were identified between the incidence of sexual and physical violence and socioeconomic and demographic factors from the census sectors in which the victims lived. Despite the decrease in the general demand for pediatric emergency assistance during COVID-19 pandemic, there was a significant increase in the number of cases of child violence, especially self-inflicted injuries due to drug intoxication in female adolescents, who are more vulnerable to self-destructive behavior during social isolation.

In summary, future studies should identify and understand the complex determinants of this phenomenon. Moreover, there is an urgent need to allocate resources for the implementation of preventive public policies, while enabling the active participation of the members of society. These measures will, in turn, protect and guarantee children and adolescents’ well-being.

## Figures and Tables

**Table 1:** Classification of socioeconomic and demographic factors in deciles.

Socioeconomic and Demographic Factors	Very Low	Low	Moderate	High	Very High
Area (km^2^)	≤0.055	≤0.101	≤0.233	≤1.055	>1.055
Demographic density (people/km^2^)	≤877	≤3.816	≤7.976	≤14.426	>14.426
Number of households	≤162	≤227	≤274	≤355	>355
EAP	≤149	≤218	≤363	≤568	>568
Absolute population	≤456	≤692	≤843	≤1.141	>1.141
Population with formal work	≤13	≤64	≤206	≤495	>495
Average income	≤3.474	≤4.108	≤4.475	≤5.906	>5.906

Source: IBGE, 2011. Numbers classified according to the statistical criteria described above. EAP = Economically active population.

**Table 2:** Full description for age.

	Average	Median	SD	CV	Q1	Q3	F	Min	Max	N	CI
Infant	0.60	1	0.50	83%	0	1	1	0	1	30	0.18
Child	4.64	4	2.21	48%	3	6	3	2	9	232	0.28
Pre-adolescent	11.86	12	1.03	9%	11	13	13	10	13	130	0.18
Adolescent	15.63	16	1.10	7%	15	17	16	14	18	217	0.15
General	9.90	11	5.47	55%	4	15	16	0	18	609	0.43

SD = Standard deviation; CV = Coefficient of variation; Q1 = First quartile; Q3 = Third quartile; F = Fashion; Min = Minimum value; Max = Maximum value; N = Sample number; CI = Confidence interval.

**Table 3:** Comparison of victim’s gender to age.

		Mean	SD	N	CI	df	*t*-Test	*p*-Value
Infant	Female	0.57	0.51	23	0.21	28	−0.687	0.498
	Male	0.71	0.49	7	0.36			
Child	Female	4.48	2.28	162	0.35	230	−1.710	0.089
	Male	5.01	2.01	70	0.47			
Pre-adolescent	Female	11.85	1.01	113	0.19	128	−0.340	0.734
	Male	11.94	1.20	17	0.57			
Adolescent	Female	15.62	1.09	169	0.16	215	−0.284	0.777
	Male	15.67	1.15	48	0.33			
General	Female	10.10	5.45	467	0.49	607	1.655	0.098
	Male	9.23	5.48	142	0.90			

SD = Standard deviation; N = Sample number; CI = Confidence interval; df = degrees of freedom.

**Table 4:** Association between the incidence of physical and sexual violence and socioeconomic and demographic factors extracted by census sectors.

Socioeconomic and Demographic Factors		Physical		Sexual		Total		*p*-Value
		N	%	N	%	N	%	
Area (km^2^)	Very low	47	22.1%	68	18.8%	115	20.00%	0.051
	Low	52	24.4%	63	17.5%	115	20.00%	
	Moderate	45	21.1%	69	19.1%	114	19.9%	
	High	37	17.4%	80	22.2%	117	20.4%	
	Very high	32	15.00%	81	22.4%	113	19.7%	
Demographic density (people/km^2^)	Very low	32	15.1%	83	23.1%	115	20.1%	0.084
	Low	39	18.4%	77	21.4%	116	20.3%	
	Moderate	46	21.7%	67	18.7%	113	19.8%	
	High	50	23.6%	63	17.5%	113	19.8%	
	Very high	45	21.2%	69	19.2%	114	20.00%	
Number of households	Very low	39	18.4%	76	21.2%	115	20.1%	0.432
	Low	40	18.9%	77	21.4%	117	20.5%	
	Moderate	49	23.1%	62	17.3%	111	19.4%	
	High	45	21.2%	70	19.5%	115	20.1%	
	Very high	39	18.4%	74	20.6%	113	19.8%	
EAP	Very low	33	15.5%	72	23.2%	105	20.1%	0.085
	Low	50	23.5%	57	18.4%	107	20.5%	
	Moderate	47	22.1%	63	20.3%	110	21.00%	
	High	34	16.00%	62	20.00%	96	18.4%	
	Very high	49	23.00%	56	18.1%	105	20.1%	
Absolute population	Very low	40	18.9%	78	21.7%	118	20.7%	0.716
	Low	44	20.8%	73	20.3%	117	20.5%	
	Moderate	46	21.7%	62	17.3%	108	18.9%	
	High	44	20.8%	76	21.2%	120	21.00%	
	Very high	38	17.9%	70	19.5%	108	18.9%	
Population with formal work	Very low	34	17.4%	62	22.5%	96	20.4%	0.251
	Low	44	22.6%	50	18.1%	94	20.00%	
	Moderate	39	20.00%	54	19.6%	93	19.7%	
	High	35	17.9%	63	22.8%	98	20.8%	
	Very high	43	22.1%	47	17.00%	90	19.1%	
Average income	Very low	40	18.9%	75	20.9%	115	20.1%	0.062
	Low	34	16.00%	80	22.3%	114	20.00%	
	Moderate	37	17.5%	77	21.4%	114	20.00%	
	High	50	23.6%	64	17.8%	114	20.00%	
	Very high	51	24.1%	63	17.5%	114	20.00%	

Source: The authors of this study. EAP = Economically active population.

## Data Availability

The data presented in the present study are available on request from the corresponding author.

## Supplementary Materials

The following are available online at www.mdpi.com/article/10.3390/ijerph181910061/s1, Figure S1: Self-inflicted injury rate in adolescents under 18 years old in relation to all visits required for general causes in the pediatric emergency room, by semester, from 2016 to 2020.
