# Supplementary figures and images for: Epidemiological Study of Violence against Children and Its Increase during the COVID-19 Pandemic
Source: Int J Environ Res Public Health. 2021 Sep 24;18(19):10061. doi: 10.3390/ijerph181910061 (PMC8507936; doi:10.3390/ijerph181910061)

## Self-inflicted Violence Rate Evolution

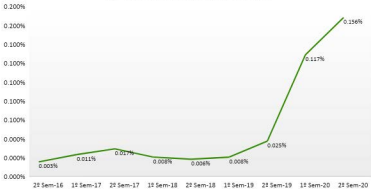

Supplement: Supplementary file 1 [file ijerph-18-10061-s001.zip › ijerph-1323540-supplementary.pdf]
